# Influence of Obtaining Conditions on Kinetics of the Initial Sintering Stage of Zirconia Nanopowders

**DOI:** 10.1186/s11671-016-1452-3

**Published:** 2016-05-04

**Authors:** Marharyta Lakusta, Igor Danilenko, Tetyana Konstantinova, Galina Volkova

**Affiliations:** Material Science Department, Donetsk Institute for Physics and Engineering (DIPE) named after O.O. Galkin of the NAS of Ukraine, Nauky av., 46, Kiev, 03680 Ukraine

**Keywords:** Zirconia nanopowders, Sintering mechanisms of ceramics, Constant rate of heating (CRH) method

## Abstract

The present paper is devoted to the problem of sintering ceramics based on yttria-stabilized zirconia (Y-TZP). In this paper, we studied the effect of two obtaining methods (co-precipitation and technical hydrolysis) on sintering kinetics of Y-TZP nanopowders. We used the constant rate of heating (CRH) method at different heating rates for determining the sintering mechanisms. The basic mechanism and activation energy (*Q*) of diffusion at the initial sintering stage were estimated using the sintering rate equations that are applicable to the CRH data. We found that nanopowder 3Y-TZP produced by the co-precipitation method (DIPE) was sintered according to the volume diffusion mechanism (*n* = 1/2) and nanopowder TZ-3Y (TOSOH) produced by the technical hydrolysis was sintered according to the grain boundary diffusion mechanism (*n* = 1/3).

## Background

Yttria-stabilized zirconia (Y-TZP) ceramics has excellent mechanical properties, such as high fracture toughness, strength, and hardness. This ceramic material is widely used for different applications [1]. The properties of Y-TZP ceramics strongly depend on the obtaining conditions of nanopowders which can control their grain size and sinterability [2].

Ceramic properties defined not only various powder-obtaining conditions but also forming and sintering methods. Last year’s several trends are in the modern production of ceramic materials: developing new methods of nanopowder synthesis; nanoparticle surface modification; and activation of sintering by dopants [1, 3–5].

The initial stage of sintering of various ceramic powders has been investigated by many researchers [1–3, 6]. Application of non-isothermal sintering methods at a constant rate of heating (CRH) plays an important role in the sintering of nanopowders. Using this method, we can examine the initial sintering stage in detail. It is important to investigate the consolidation processes exactly, which are connected with peculiar properties of the sintering of nanoparticles with a small grain size and high specific surface area. WS Young and IB Cutler [7] have investigated the initial sintering behavior in Y-TZP under CRH. J Wang and R Raj have estimated the activation energies at the initial sintering stage of Y-TZP and Al_2_O_3_/Y-TZP composite by the CRH method [6, 8].

The main goal of this paper is investigation of the influence of different nanopowder-obtaining conditions on sintering kinetics of ceramic nanocomposites based on Y-TZP. It is especially important to clarify the effect of various nanopowder-obtaining conditions on the sintering process. This data will ensure possibility to control the sintering process of Y-TZP ceramics for producing new ceramic nanocomposites.

## Methods

### Samples Preparation

We used two kinds nanopowders: TZ-3Y (TOSOH, Tokyo, Japan) containing 3 mol % Y_2_O_3_ manufactured by hydrolysis method and 3Y-TZP containing 3 mol % Y_2_O_3_ (DIPE of the NASU, Ukraine) produced by co-precipitation method.

The preparation technique according to the manufacture’s data consists several stages: ZrOCl_2_*8H_2_O, YCl_3_ → technical hydrolysis process → drying → calcinations → mechanical milling (48 hours) → spray-drying (hot gas) → nanopowder TZ-3Y (TOSOH).

Mechanical effects are used in technologies based on conventional thermal solid-phase synthesis, mixing the components and increasing the contact surface between the solid particles. Agglomeration degree depends on nanopowder-obtaining method [9]. Mechanical activation in the mills is often used for reducing the aggregation degree and the activation of the particle surface [9].

The preparation technology of the second powder has the following stages: initial salt dissolution ZrOCl_2_*8H_2_O, YCl_3_ → co-precipitation → drying → calcination → nanopowder ZrO_2_-3 mol % Y_2_O_3_ (3Y-TZP, DIPE) without mechanical milling and spray-drying. All the used chemicals have chemical purity. At first, appropriate amounts of Y_2_O_3_ were dissolved in nitric acid; then, the zirconium and yttrium salts are mixed via a propeller stirrer for 30 min and subsequently added to an aqueous solution of the precipitant (25 % NH_4_OH) with constant stirring. Sediments were mixed for 1 h at room temperature at a pH of 9. The sediments were then repeatedly washed and filtered with distilled water. For chloride salts, washing was carried out until a negative test for C1^−^ ions with a silver nitrate solution. After washing and filtration, the hydrogel was dried in a microwave furnace. The calcination of dried zirconia hydroxides was carried out in resistive furnaces at temperature 1000 °C for 2 h.

The powders obtained after calcination were characterized by X-ray diffraction (XRD) employing a DRON-3 diffractometer with Cu-K-α radiation. Fitting and analysis of the XRD curves were made by Powder Cell software for Windows version 2.4. The powders were also studied by transmission electron microscopy (TEM) (JEM 200A, JEOL, and Japan), and the observed average particle size was compared with the value obtained by XRD. Reliable data were obtained by analyzing data from 30 TEM fields for both powders.

Further, all the powders were pressed uniaxially into cylinder specimens with the following dimensions: diameter 6 mm, height 15–17 mm. After that, the powder compacts were treated by a high hydrostatic pressure of 300 MPa.

The shrinkage data of the sintering powder compacts was obtained using a dilatometer (NETZSCH DIL 402 PC). The dilatometer was calibrated using a standard sample of Al_2_O_3_. Measurements of shrinkage by the CRH method were carried out in the range from room temperature to 1500 °C with different heating rates of 2.5, 5, 10, 20 °C/min. Upon reaching the temperature of 1500 °C, the samples were cooled at a constant rate. Thermal expansion of each sample was corrected with the cooling curve by the method described in [9]. It was confirmed that the shrinkage proceeded isotropically. The density of the sintered samples was measured using the Archimedes method.

### The Analytical Method for the Determination of the Sintering Mechanism at the Initial Sintering Stage

The diffusion mechanism and activation energy of diffusion at the initial sintering stage were determined by the same analytical method as that in the papers [1, 2, 4, 5]. The initial sintering stage is not more than 4 % of relative shrinkage. In this temperature ranges, interparticle contacts begin to form only and grain growth is negligible. Assuming isotropic shrinkage of the samples, the density *ρ* (T) at a given temperature *T* is given by the following equation [6]:

1 $$ \rho (T)={\left(\frac{L_{\mathrm{f}}}{L(T)}\right)}^3{\rho}_{\mathrm{f}} $$

where *L*_f_ is the final length; *L*(*T*), the length of the sample at temperature *T*; and ρ_f_, the final density of the sample measured by hydrostatic method. For investigations, the relative shrinkage no greater than 4 % was selected.

The diffusion mechanism and activation energies were determined by the analytical method described in [4]. Sintering rate at the initial sintering stage is expressed by the equation:

2 $$ \frac{d}{dt}\left[{\left(\frac{\varDelta L}{L_0}\right)}^{\frac{1}{n}}\right]=\frac{K\gamma \varOmega D}{kTaP} $$

*ΔL* = (*L*_0_−*L*) is the change in length of the specimen; *K*, the numerical constant; *Ω*, the atomic volume; *D*, the diffusion coefficient; *γ*, the surface energy; *t*, the time; *T*, the temperature; *k*, the Boltzmann constant; *a*, the particle radius; and parameters *n* and *p*, the order depending on diffusion mechanism. Since value ${\left(\frac{\varDelta L}{L_0}\right)}^{\frac{1}{n}}$ may be expressed as a density function *F*′ (*ρ*), we obtain:

3 $$ {\left(\frac{\varDelta L}{L_0}\right)}^{\frac{1}{n}}={F}^{\prime}\left(\rho \right) $$

The equation for the sintering rate at the initial stage in the CRH process can be separated into temperature-dependent, grain size-dependent, and density-dependent quantities as follows (J Wang and R Raj) [6, 8, 10] :

4 $$ T\cdot c\frac{d\rho }{dT}=\frac{1}{F^{\prime}\left(\rho \right)}\cdot \frac{K\gamma \varOmega D}{kTaP}\cdot \exp \left(-\frac{Q}{RT}\right) $$

The following sintering rate equation that is applicable to CRH data is derived from Eq. (2)

5 $$ \ln \left[T\left(\frac{dT}{dt}\right)\left(\frac{d\rho }{dT}\right)\right] = \hbox{-} \frac{Q}{RT} + \alpha \left(n,p\right) $$

6 $$ \alpha \left(n,p\right) = \ln \left[f\left(\rho, n\right)\right] + \ln\ \left[\frac{K\gamma \varOmega {D}_0}{k}\right]\ \hbox{-}\ p \ln $$

Here, *Q* is the activation energy; *R* is the gas constant; *f* (*ρ*, *n*) is the density function that depends on *n*; *c* = *dT*/*dt* is the heating rate; and *D*_0_ is the pre-exponential term defined as *D* = *D*_0_exp(−*Q*/*RT*).

Equation (4) corresponds to the sintering rate equation derived by J Wang and R Raj. Using the slope *S*_1_ of the Arrhenius-type plot of ln[*T*(*dT*/*dt*)(*dρ*/*dT*)] against 1/*T* at the same density, the *Q* is expressed as

7 $$ Q = \hbox{-}\ R{S}_1 $$

From Equation (4), Yang and Cutler derived the following sintering rate equation [7]:

8 $$ \frac{d\left(\varDelta L/{L}_0\right)}{dT}=\left(\frac{K\gamma \varOmega {D}_0R}{kaPcQ}\right)\cdot \left(\frac{nQ}{R{T}^{2-n}}\right)\cdot \exp \left(-\frac{nQ}{RT}\right) $$

Using the slope *S*_2_ of the Arrhenius-type plot of ln[*T*^2 − *n*^*d*(*ΔL*/*L*_0_)/*dT*] against 1/*T*, we found:

9 $$ nQ = - R{S}_2 $$

From Equations (7) and (9), we found the order of diffusion mechanism:

10 $$ n = \frac{S_2}{S_1} $$

Here, *n* is in the range of 0.31–0.50. According to two-sphere shrinkage models proposed by several researchers [2, 10], the *n* value ranges of grain boundary diffusion (GBD) and volume diffusion (VD) are 0.31–0.33 and 0.40–0.50, respectively.

## Results and Discussion

**Fig. 1:**
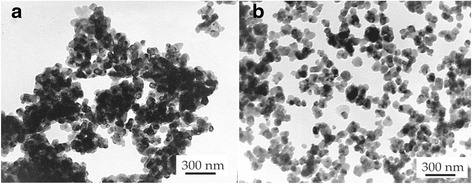
Transmission electron microscopy (TEM) images of nanopowder structures. **a** 3Y-TZP (DIPE). **b** TZ-3Y (TOSOH)

**Fig. 2:**
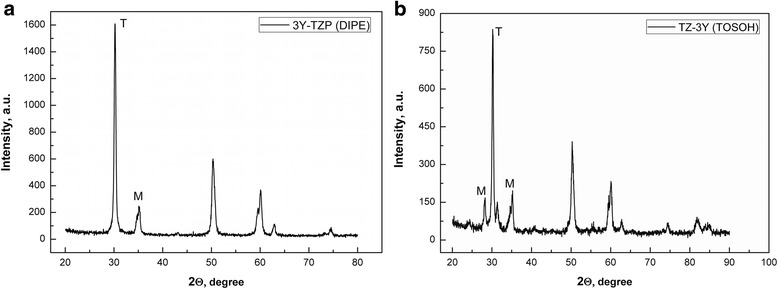
X-ray diffraction pattern of **a** 3Y-TZP (DIPE) and **b** TZ-3Y (TOSOH)

In Fig. 1, we can see the TEM images of nanopowder structure: (a) 3Y-TZP (DIPE) and (b) TZ-3Y (TOSOH). The initial characteristics of nanopowders TZ-3Y (TOSOH) and 3Y-TZP (DIPE) are given in Table 1. As it can be seen, after calcinations of 3Y-TZP (DIPE), we obtained the powder with practically equal particle sizes 31 ± 1.5 nm for 3Y-TZP (DIPE) and 27 ± 1.4 nm for TZ-3Y (TOSOH). The agglomeration degree of the 3Y-TZP (DIPE) nanopowder was larger in comparison with that of TZ-3Y (TOSOH). This means that the powders have different sinterability, what may be due to the different surface states of nanopowders obtained under different conditions. It should be noted that agglomerates in the 3Y-TZP (DIPE) nanopowder are soft and easily destroyed under mechanic and high hydrostatic pressure effects [9]. In comparison with the 3Y-TZP (DIPE) powder, TZ-3Y (TOSOH) was processed by mechanical milling, i.e., their surface was strongly activated. As shown in Fig. 2, the amounts of monoclinic phase in the nanopowder TZ-3Y (TOSOH) are slightly larger than those in 3Y-TZP (DIPE). It is also due to the milling of powder TZ-3Y (TOSOH).

**Table 1:** The initial characteristics of nanopowders TZ-3Y (TOSOH) and 3Y-TZP (DIPE)

Powders	S_BET_ (m^2^/g)	˂D_RCS_˃ (nm)	The phase composition
3Y-TZP (DIPE)	15	31	7 % M + T
TZ-3Y (TOSOH)	15	27	18 % M + T

*M* monoclinic phase, *T* tetragonal phase

**Fig. 3:**
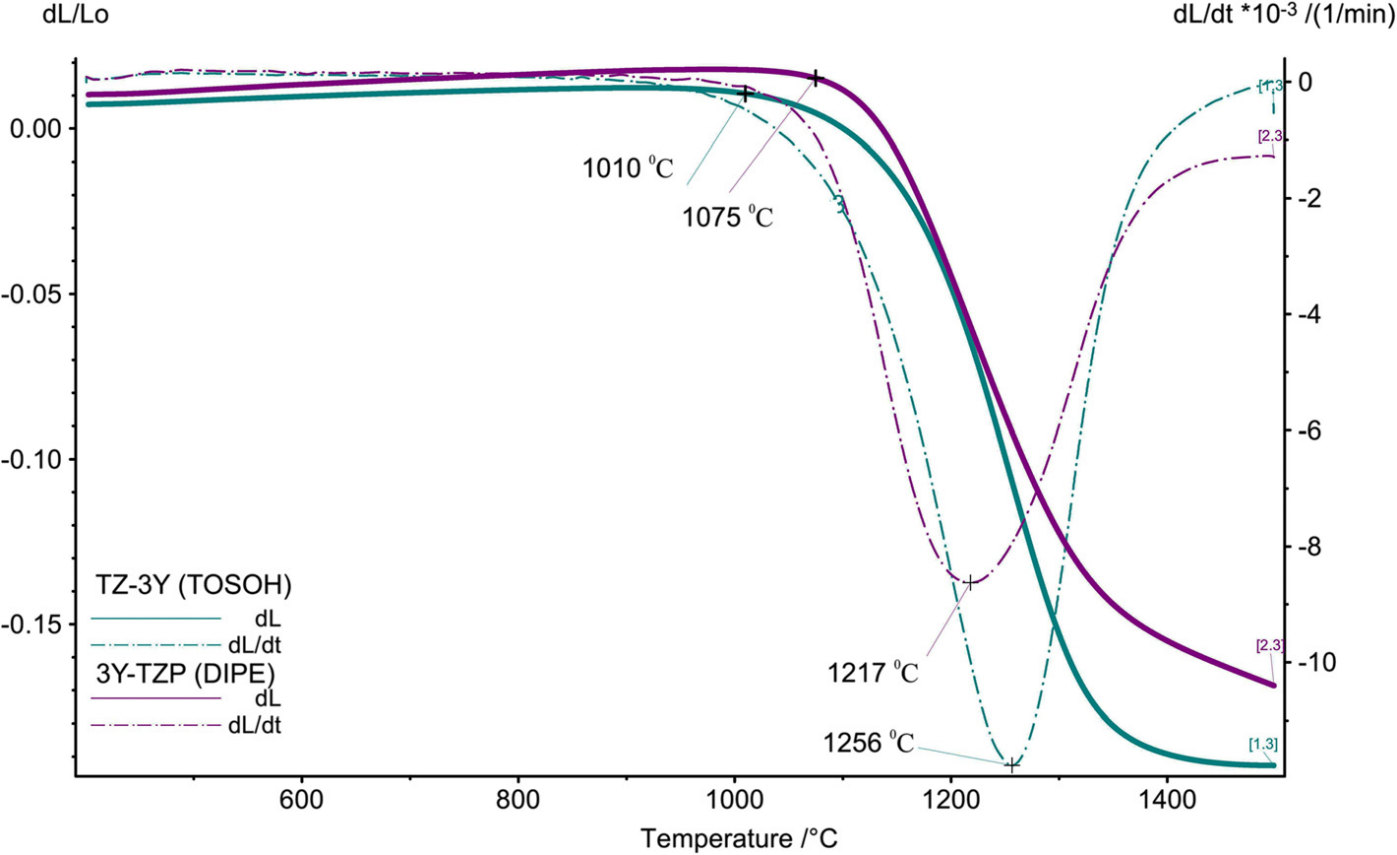
Temperature dependence of shrinkage and the shrinkage rate for the samples 3Y-TZP (DIPE) and TZ-3Y (TOSOH) at heating rate 10 °C/min

Figure 3 shows the temperature dependence of the relative shrinkage (*dL*/*L*_0_) and shrinkage rate (*ΔL*/*dt*) of the samples 3Y-TZP (DIPE) and TZ-3Y(TOSOH) at a heating rate of 10 °C/min from room temperature to 1500 °C. As it can be seen, the onset sintering temperatures of shrinkage of these samples are different. In samples TZ-3Y (TOSOH), shrinkage begins earlier at 1010 °C, then shrinkage of samples 3Y-TZP (DIPE) begins at 1075 °C. The highest rate of shrinkage was achieved at temperatures of 1217 and 1256 °C in samples 3Y-TZP and TZ-3Y (TOSOH), respectively. It can be seen that the shrinkage rate of sample 3Z-3Y (DIPE) is more intensive than that of sample TZ-3Y (TOSOH).

**Fig. 4:**
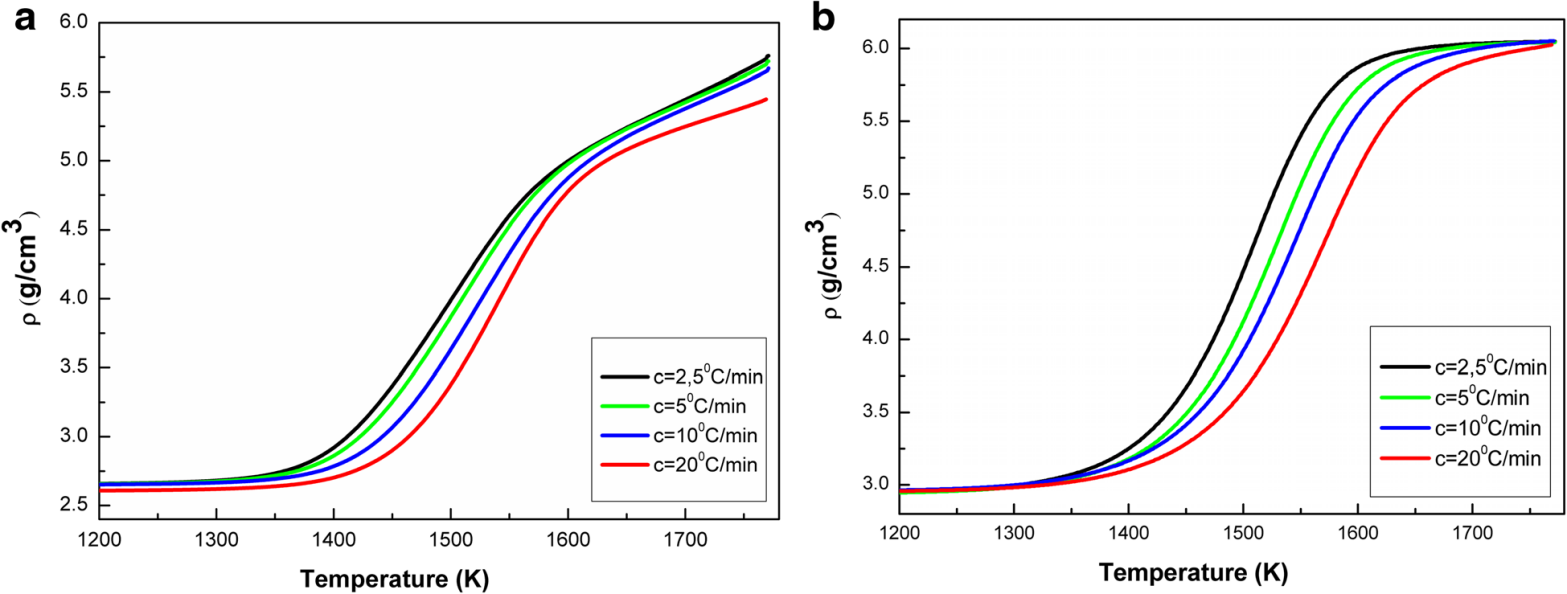
Temperature dependence of density of powders **a** 3Y-TZP (DIPE) and **b** TZ-3Y (TOSOH) at different heating rates

**Fig. 5:**
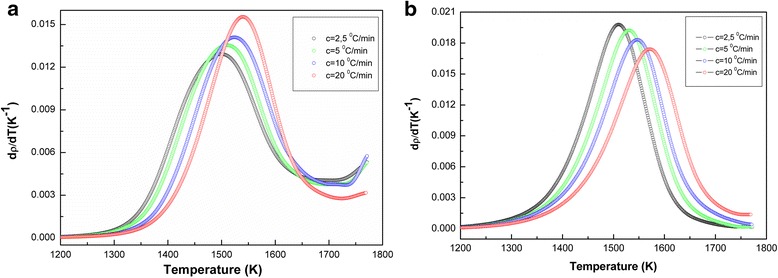
Temperature dependence of densification rate of samples **a** 3Y-TZP (DIPE) and **b** TZ-3Y (TOSOH) at different heating rates

Figure 4a, b shows the density change of the samples with increasing the sintering temperature from 1300 to 1800 K. In Fig. 5, we can see the temperature dependence of the densification rate (*dρ*/*dT*) for the samples (a) TZ-3Y (TOSOH) and (b) 3Y-TZP (DIPE) at different heating rates of 2.5, 5, 10, and 20 °C/min. For samples (a) TOSOH TZ-3Y and (b) 3Y-TZP (DIPE), densification rate curves have shifted to higher temperatures with increasing heating temperature. Densification rate of the samples is different at the same sintering temperatures. In sample TZ-3Y (TOSOH), densification rate is much higher than that in sample 3Y-TZP (DIPE).

**Fig. 6:**
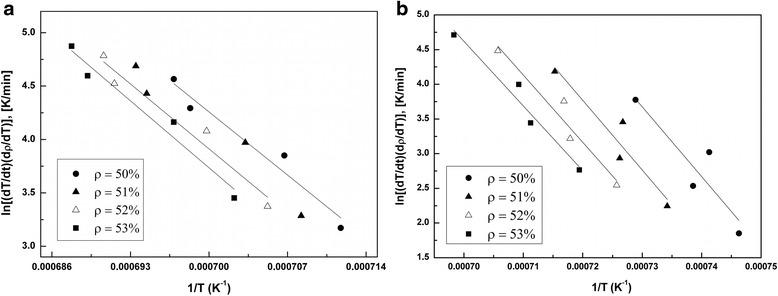
Arrhenius-type plots of samples **a** 3Y-TZP (DIPE) and **b** TZ-3Y (TOSOH)

The diffusion mechanism of sintering samples TZ-3Y (TOSOH) and 3Y-TZP (DIPE) was determined by the method described in articles [1, 4, 5, 10] using equations (4, 5, 6, 7, 8, and 9) and Arrhenius-type plots presented in Fig. 6. The obtained activation energy and the order of diffusion mechanisms (*n* = 0.33–0.51) for the samples are shown in Table 2.

**Table 2:** Activation energy and the order of diffusion mechanisms for the samples TZ-3Y (TOSOH) and 3Y-TZP (DIPE)

Powders	Number	*Q* (kJ/mol)
TZ-3Y (TOSOH)	1/3	840 ± 40
3Y-TZP (DIPE)	1/2	667 ± 40

As shown in Table 2 we found that the sample 3Y-TZP (DIPE) was sintered according to the VD mechanism (*n* = 1/2). And the sample TZ-3Y (TOSOH) was sintered by the grain boundary diffusion (GBD) mechanism (*n* = 1/3). We can conclude that in samples 3Y-TZP (DIPE), mass transfer process is more intensive than in samples TZ-3Y (TOSOH), which was confirmed by higher sintering rate at the initial stage of sintering.

## Conclusions

In the present study, the authors investigated the kinetics of the initial sintering stage of Y-TZP nanopowders manufactured by the hydrolysis method with final processing in mill and co-precipitation method. We used the analytical method applicable to the experimental data obtained at the constant rate of heating (CRH) and found that nanopowder-obtaining conditions significantly affect mass transfer mechanisms at sintering, as well as the activation energy of sintering and the densification rate of zirconia nanopowders. In case of the 3Y-TZP (DIPE) nanopowder obtained by co-precipitation, the sintering occurs predominantly according to the volume diffusion VD mechanism, and in case of the TZ-3Y (TOSOH) nanopowder prepared by the method of technical hydrolysis, the sintering occurs predominantly according to the grain boundary diffusion GBD mechanism.

Since the technical hydrolysis and co-precipitation process are similar and sample characteristics, such as surface area, particle size, and phase structure of nanopowders, are nearly the same, however, predominant diffusion mechanisms of sintering at the initial stage appeared different. Strong surface activation predetermined implementation of grain boundary mechanism at the initial stage of sintering, which requires higher activation energy. It is known that a volume sintering mechanism is preferred due to the relatively low activation energy and the possibility of reducing the sintering temperature of ceramics.

## Abbreviations

3Y-TZP: 3 mol % yttria-stabilized tetragonal zirconia polycrystal
CRH: constant rate of heating
DIPE: Donetsk Institute for Physic and Engineering
GBD: grain boundary diffusion
NAS: National Academy of the Science
TEM: transmission electron microscopy
TZ-3Y (TOSOH): 3 mol% yttria-stabilized tetragonal zirconia (Tosoh Corporation Production)
VD: volume diffusion
XRD: X-ray diffraction
